# Tissue‐specific activation of *DOF11* promotes rice resistance to sheath blight disease and increases grain weight via activation of *SWEET14*

**DOI:** 10.1111/pbi.13489

**Published:** 2020-11-14

**Authors:** Pyol Kim, Cai Yun Xue, Hyon Dok Song, Yue Gao, Lu Feng, Yuhua Li, Yuan Hu Xuan

**Affiliations:** ^1^ Key Laboratory of Saline‐alkali Vegetation Ecology Restoration Ministry of Education (Northeast Forestry University) Harbin China; ^2^ College of Plant Protection Shenyang Agricultural University Shenyang China

**Keywords:** SWEET14, yield, sheath blight disease, DOF11, rice

Sugar will eventually be exported transporter (SWEET) is a family of sugar transporters that plays a critical role during host and pathogen interaction (Bezrutczyk *et al*., 2018). In rice, *SWEET11/Xa13*, *SWEET13/Xa25* and *SWEET14* were identified as targets of *Xanthomonas oryzae* pv. *oryzae* effectors (Antony *et al*., 2010; Hutin *et al*., 2015; Yang *et al*., 2006). However, the role of SWEET genes in rice and *Rhizoctonia solani*, the causative agent of sheath blight disease (ShB), is largely unknown. Our previous transcriptome data showed that *SWEET14* expression is sensitive to *R. solani* infection (De Peng Yuan, 2020). qPCR results verified that *R. solani* infection dramatically induces *SWEET14* expression along with a pathogen‐related protein *PBZ1* (Figure 1a). Furthermore, CRISPR/Cas9‐mediated genome editing mutants and overexpression lines were generated, and sequencing of the genomic DNA indicated that the fifth exon of *SWEET14* in *sweet14‐1* and *sweet14‐2* mutants had a 2‐bp deletion and a 1‐bp insertion, respectively (Figure 1b). The expression of *SWEET14* was slightly higher in *sweet14* mutants while significantly higher in the overexpressors, *SWEET14 OX* (*#1*, *#2*) compared to the wild‐type plants (Figure 1c). *R. solani* AG1‐IA inoculation demonstrated that *sweet14* mutants were more susceptible while *SWEET14 OX* lines were less susceptible to ShB (Figure 1d). These results suggest that SWEET14 may reduce sugar content in the apoplasm to inhibit *R. solani* growth (Figure 1e). However, *SWEET14 OX* resulted in a dwarf phenotype, reduced 1,000‐grain weight and grain number per panicle, and normal tiller growth, while *sweet14* mutants maintained normal growth and yield production (Figure 1f–i).

**Figure 1:**
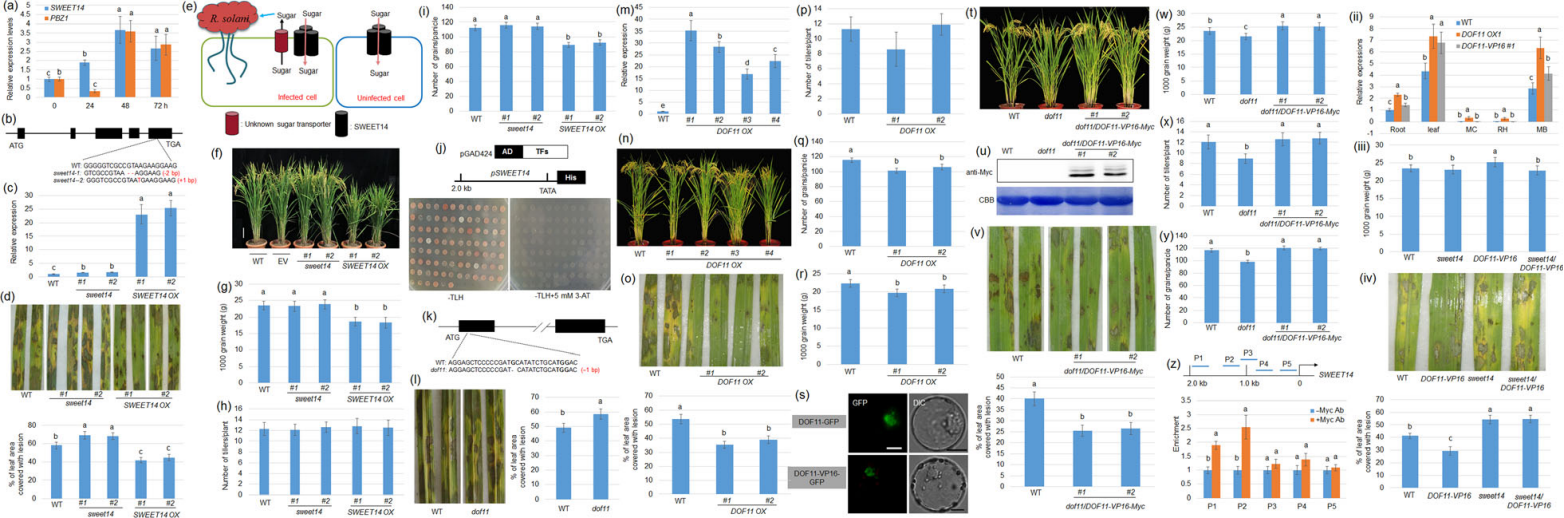
DOF11 activates *SWEET14* to regulate rice production and resistance to ShB. (a) *PBZ1* and *SWEET14* expression levels were examined after 0, 24, 48 and 72 h of *R. solani* inoculation using qRT‐PCR. Data indicate the average ± SE (*n* = 3). (b) Genomic structure and mutant information of *SWEET14*. Black boxes and lines indicate exons and introns, respectively. The sequences below the fifth exon indicate wild‐type (WT) and CRISPR/Cas9‐induced genome editing mutant sequences. (c) Expression of *SWEET14* in WT, *sweet14* (*#1*, *#2*) and *SWEET14 OX* (*#1*, *#2*) was examined. Data indicate the average ± SE (*n* = 3). (d) Leaves from the WT, *sweet14* (*#1*, *#2*) and *SWEET14 OX* (*#1*, *#2*) were inoculated with *R. solani*. The lesion areas on the leaf surfaces were examined. Data indicate the average ± SE (*n* > 10). (e) The model of SWEET14 action in rice defence to *R. solani*. (f) Three‐month‐old WT, empty vector (EV), *sweet14* (*#1*, *#2*) and *SWEET14 OX* (*#1*, *#2*) plants. (g) One‐thousand‐grain weight, (h) number of tillers per plant and (i) number of grains per panicle from WT, *sweet14* (*#1*, *#2*) and *SWEET14 OX* (*#1*, *#2*). Data indicate the average ± SE (*n* > 10). (j) The library for yeast one‐hybrid was constructed in pGAD42, and 2‐kb of *SWEET14* promoter was cloned into pHISi vector with HIS as the reporter gene. The yeast cells co‐transformed with *pSWEET14‐His* and library DNA were grown on SD media (‐Try, ‐Leu and –His) or the same medium containing 5 mM 3‐amino‐1,2,4‐triazole (3AT), a competitive inhibitor of HIS3. (k) Genomic structure and mutant information of *dof11*. Black boxes and lines indicate exons and introns, respectively. The sequences below the first exon indicate WT and CRISPR/Cas9‐induced genome editing mutant. (l) Leaves from the WT and *dof11* were inoculated with *R. solani* AG1‐IA. The lesion areas on the leaf surfaces were examined. Data indicate the average ± SE (*n* > 10). (m) The expression of *DOF11* in WT and *DOF11 OX* (*#1*‐*#4*) was examined using qRT‐PCR. Data indicate the average ± SE (n = 3). (n) Three‐month‐old WT and *DOF11 OX* (*#1*‐*#2*) plants were photographed. (o) Leaves from the WT and *DOF11 OX* (*#1*, *#2*) at the time of inoculated with *R. solani* and after infection. The lesion areas on the leaf surfaces were examined. Data indicate the average ± SE (*n* > 10). (p) The number of tillers per plant, (q) number of grains per panicle, (r) one‐thousand‐grain weight from WT and *DOF11 OX* (*#1*, *#2*). Data indicate the average ± SE (*n* > 10). (s) DOF11‐GFP and DOF11‐VP16‐GFP localization in the protoplast cells. GFP and bright‐field channels were evaluated. Scale bar = 20 µm. (t) Three‐month‐old WT, *dof11* and *dof11/DOF11‐VP16‐Myc* (*#1*, *#2*). (u) Western blot analysis was performed to detect DOF11‐VP16‐Myc levels using anti‐Myc antibody in WT, *dof11* and *dof11/DOF11‐VP16‐Myc* plants (*#1*, *#2*). Coomassie brilliant blue (CBB) staining was used as the loading control. (v) Leaves from the WT and *dof11/DOF11‐VP16‐Myc* were inoculated with *R. solani*. (q) The lesion areas on the leaf surfaces were examined. Data indicate the average ± SE (*n* > 10). (w) One‐thousand‐grain weight, (x) number of tillers per plant, and (y) number of grains per panicle from WT, *dof11*, and *dof11/DOF11‐VP16‐Myc* (*#1*, *#2*). Data indicate the average ± SE (n> 10). (z) Schematic diagram indicating the location of the probes (P1‐P5) used for chromatin immunoprecipitation (ChIP) assays in *SWEET14* promoter. Relative ratios of immunoprecipitated DNA to input DNA were determined by qPCR. Input DNA was used to normalize the data. −Ab or +Ab: Myc antibody. Error bars represent ± SE (*n* = 3). (o) One‐thousand‐grain weight of WT, *dof11* and *dof11/DOF11‐VP16‐Myc* (*#1*, *#2*). Data indicate the average ± standard error (SE) (*n* > 10). (ii) *SWEET14* expression was examined in the root, leaf, mesophyll cells (MC), root hairs (RH) and mature booting stage (MB) from WT, *DOF11 OX1* and *dof11/DOF11‐VP16‐Myc #1* plants using qRT‐PCR. Data indicate the average ± SE (n = 3). (iii) One‐thousand‐grain weight of WT, *sweet14*, *DOF11‐VP16* and *sweet14/DOF11‐VP16* plants. Data indicate the average ± SE (*n* > 10). (iv) Leaves from the WT, *sweet14*, *DOF11‐VP16* and *sweet14/DOF11‐VP16* plants were inoculated with *R. solani*. The lesion areas on the leaf surfaces were examined. Data indicate the average ± SE (*n* > 10). Different letters above the bars denote statistically significant differences (*P < *0.05).

Further, the transcription factors that directly activate *SWEET14* were screened using 2‐kb of *SWEET14* promoter via yeast one‐hybrid assay (Figure 1j). Among the transcription factors isolated, DOF11 was further examined. A previous report indicated that DOF11 modulates sugar transport in rice (Wu *et al*., 2018). *DOF11* genome editing mutant showed 1‐bp deletion in the first exon and exhibited higher susceptibility to ShB (Figure 1k, l). Furthermore, *DOF11* overexpressors (OXs) (Figure 1m,n) were less susceptible to *R. solani* than wild‐type plants (Figure 1o). *DOF11 OX* developed similar tiller numbers, but its 1,000‐grain weight and grain number per panicle were reduced compared with wild‐type (Figure 1p–r). *DOF11* overexpression increased rice resistance to ShB, but reduced yield production. Therefore, the VP16, a transcriptional activation domain (Li *et al*., 2013), was fused to DOF11 in transgenic plants. DOF11‐GFP and DOF11‐VP16‐GFP were localized in the nucleus (Figure 1s). The *DOF11‐VP16‐Myc* expression under the control of 2.0 kb *DOF11* promoter rescued the *dof11* semi‐dwarf phenotype (Figure 1t). DOF11‐VP16‐Myc protein expression was detected using Western blot analysis (Figure 1u). *R. solani* inoculation showed that *dof11/DOF11‐VP16‐Myc* plants (*#1*, *#2*) were less susceptible to ShB compared to wild‐type (Figure 1v). Further examination showed that *dof11* mutants exhibited decreased 1,000‐grain weight, tiller number per plant and grain number per panicle while the *dof11/DOF11‐VP16‐Myc* plant values increased in all the phenotypes, except tiller number (Figure 1w–y). A chromatin immunoprecipitation (ChIP) assay was performed using *DOF11‐VP16‐Myc* transgenic plant calli with an anti‐Myc antibody. The results showed that DOF11‐VP16‐Myc bound to the P1 and P2 regions, but not to the P3‐P5 fragments of *SWEET14* promoter (Figure 1z).

*SWEET14* expression test in wild‐type, *DOF11 OX1* and *dof11/DOF11‐VP16‐Myc #1* showed that *SWEET14* level was higher in the root, leaf and mature booting stage in *DOF11 OX1* and *dof11/DOF11‐VP16‐Myc #1* compared with wild‐type plants, and higher in the root and at the mature booting stage of *DOF11 OX* than in *dof11/DOF11‐VP16‐Myc #1*. However, the ectopically expressed *SWEET14* in mesophyll cells and root hairs of *DOF11 OX* were not detected in corresponding tissues of *DOF11‐VP16‐Myc #1* (Figure 1ii), suggesting tissue‐specific activation of *SWEET14* by DOF11‐VP16. To analyse whether the increase of yield and resistance in *DOF11‐VP16* was via activation of *SWEET14* transcription, genetic combinations between *sweet14* and *DOF11‐VP16* plants were generated. Investigation of the yield showed that the 1,000‐grain weight of *DOF11‐VP16* was higher compared with wild‐type, *sweet14* and *sweet14/DOF11‐VP16* (Figure 1iii). Next, *R. solani* AG1‐IA inoculation results showed that *DOF11‐VP16* was less susceptible to ShB than wild‐type, *sweet14* and *sweet14/DOF11‐VP16* (Figure 1iv).

Taken together, our analyses revealed that *SWEET14*, a sugar transporter, positively regulates rice resistance to ShB. However, non‐specific transport of sugar by overexpression of *SWEET14* significantly reduced yield production, suggesting that SWEET14 plays a role in both yield production and defence. DOF11 is identified as a direct transcriptional regulator of *SWEET14*, with *DOF11* overexpression increased resistance to ShB but reduced yield production. Interestingly, tissue‐specific activation of DOF11 by fusion of VP16 increased both yield production and resistance to ShB. Expression, genetic and pathological analyses suggest that tissue‐specific activation of DOF11 simultaneously increases yield production and improves resistance to ShB, possibly partially through activation of *SWEET14*.

## Authors’ contributions

PK, CYX, YHL and YHX designed the experiments. PK, CYX and HDS performed the experiments. YG and LF manipulated plant materials. PK, YHL and YHX analysed data. YHL and YHX wrote the manuscript. All authors read and approved the final manuscript.
